# Metal–Dithiolene Bonding Contributions to Pyranopterin Molybdenum Enzyme Reactivity

**DOI:** 10.3390/inorganics8030019

**Published:** 2020-03-05

**Authors:** Jing Yang, John H. Enemark, Martin L. Kirk

**Affiliations:** 1 Department of Chemistry and Chemical Biology, The University of New Mexico, MSC03 2060, Albuquerque, NM 87131-0001, USA;; 2 Department of Chemistry Biochemistry, University of Arizona, Tucson, AZ 85721, USA;

**Keywords:** metal-dithiolene, pyranopterin molybdenum enzymes, fold-angle, tungsten enzymes, electronic structure, pseudo-Jahn-Teller effect, thione, molybdenum cofactor, Moco

## Abstract

Here we highlight past work on metal-dithiolene interactions and how the unique electronic structure of the metal-dithiolene unit contributes to both the oxidative and reductive half reactions in pyranopterin molybdenum and tungsten enzymes. The metallodithiolene electronic structures detailed here were interrogated using multiple ground and excited state spectroscopic probes on the enzymes and their small molecule analogs. The spectroscopic results have been interpreted in the context of bonding and spectroscopic calculations, and the pseudo-Jahn-Teller effect. The dithiolene is a unique ligand with respect to its redox active nature, electronic synergy with the pyranopterin component of the molybdenum cofactor, and the ability to undergo chelate ring distortions that control covalency, reduction potential, and reactivity in pyranopterin molybdenum and tungsten enzymes.

## 1. Introduction

**Figure 1.**
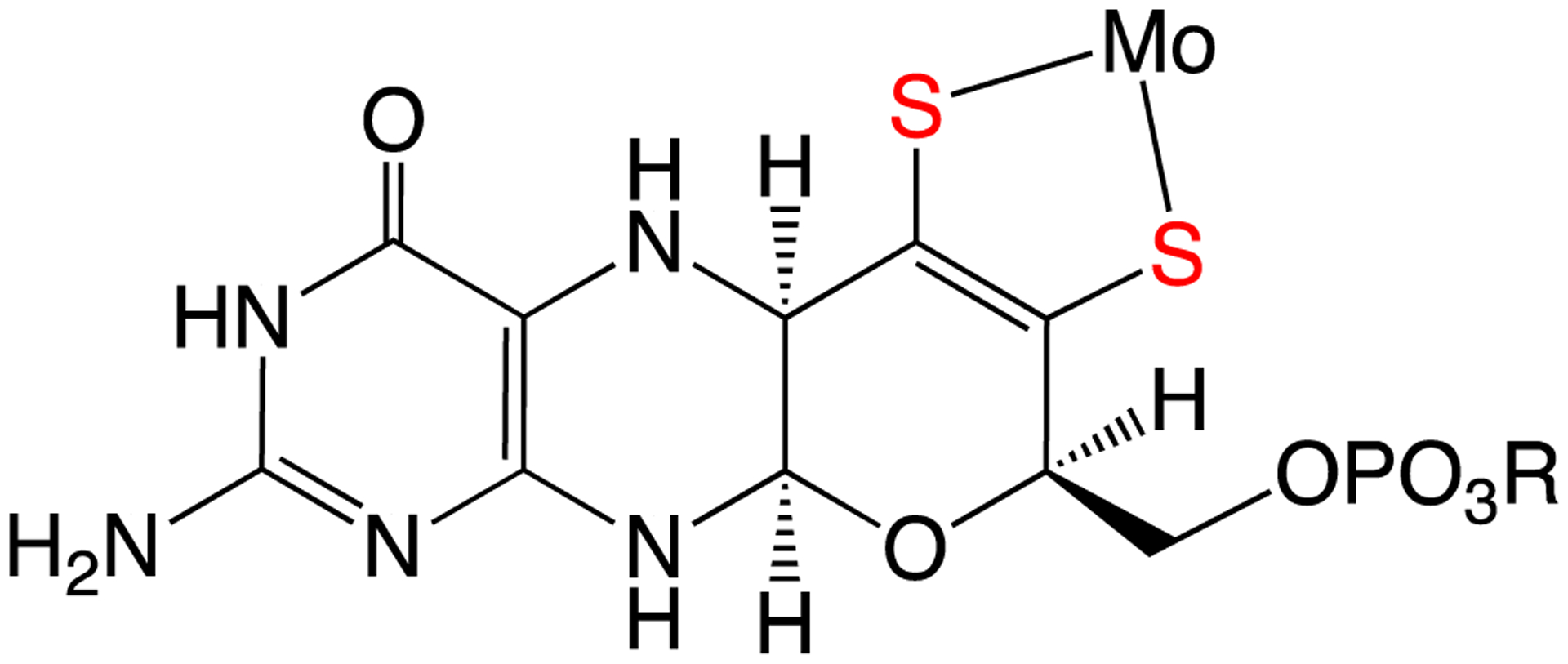
The reduced tetrahydro form of the pyranopterin dithiolene (PDT) coordinated to Mo in the molybdenum cofactor (Moco). In the enzymes, the Mo ion can redox cycle between the Mo^IV^, Mo^V^, and Mo^VI^ oxidation states.

It is now well-established that all known molybdenum-containing enzymes [1–3], with the sole exception of nitrogenase, contain a common pyranopterin dithiolene (PDT) (Figure 1) organic cofactor (originally called molybdopterin (MPT)), which coordinates to the Mo center of the enzymes through the sulfur atoms of the dithiolene fragment. To date, the PDT component [4] of the molybdenum cofactor (Moco) is the only known occurrence of dithiolene ligation in biological systems. This cofactor is also found in anaerobic tungsten enzymes, and it may be one of the most ancient cofactors in biology [5]. The study of metal-dithiolene compounds (metallodithiolenes) has undergone a recent renaissance, with their synthesis, geometric structure, spectroscopy, bonding, and electronic structure having been recently highlighted [4,6–20]. Here, we briefly review the discovery of metallodithiolene compounds [13,21]. This history is followed by a more extensive discussion of key investigations into the myriad roles of the dithiolene ligands in the structure, bonding and reactivity of metal compounds, using multiple spectroscopic techniques, as well as theoretical calculations. Throughout this review, the key implications of these results for Mo and W enzymes are discussed.

**Figure 2.**
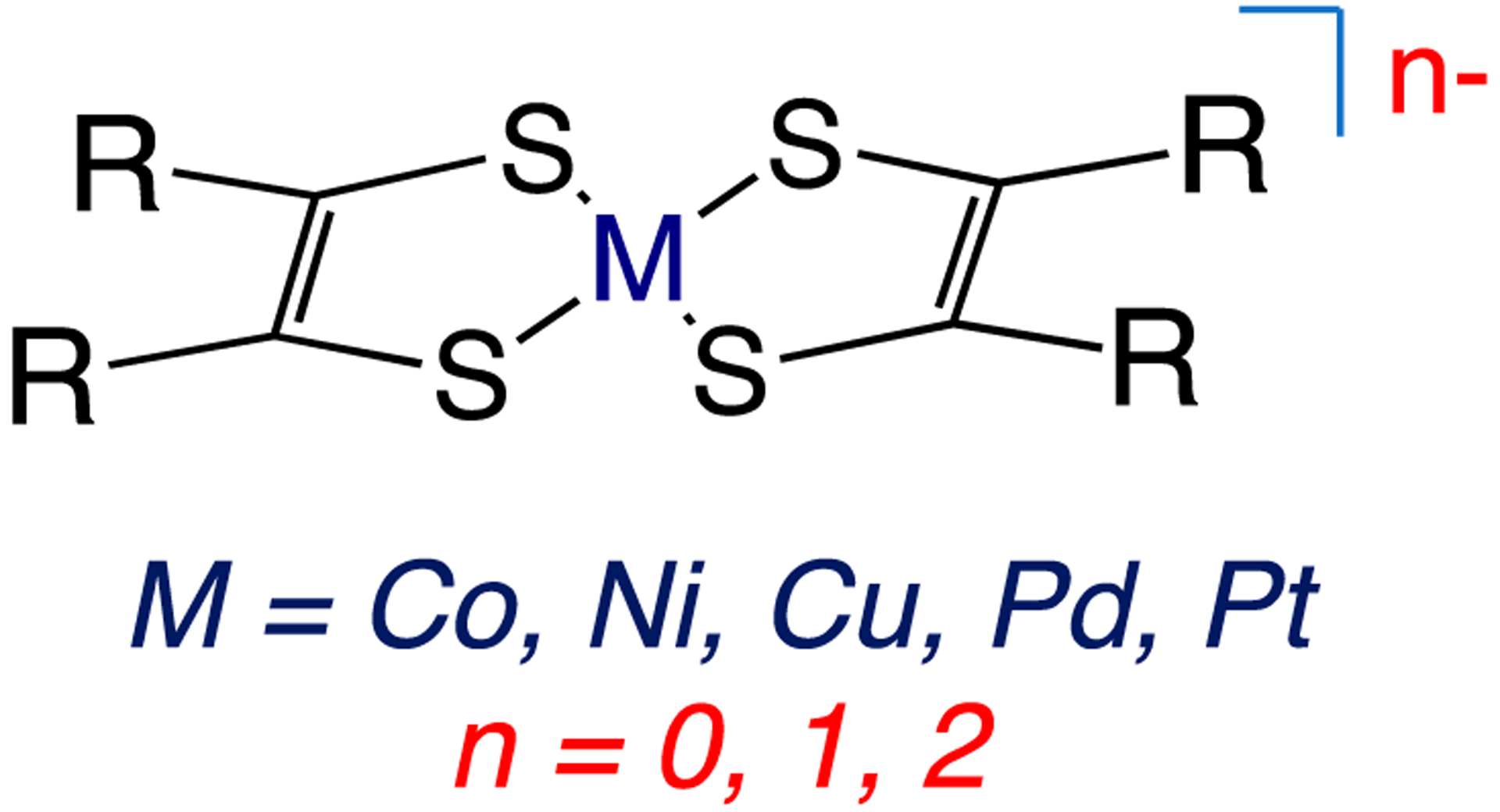
Square planer metallodithiolene complexes. R = CN, CH_3_, Ph, CF_3_.

In the early 1960s, several research groups reported intensely colored square planar metal complexes with chelating sulfur-donor ligands that could stabilize metal compounds in a range of formal oxidation states related by one-electron oxidation-reduction (i.e., redox) reactions (Figure 2) [22–24]. McCleverty gave these novel ligands the general name “dithiolene” in order to emphasize their delocalized electronic structures [25]. These ligands are also described as being “non-innocent” due to the participation of the dithiolene ligands in the multiple one-electron reactions of their metal complexes and the inability to assign a specific oxidation state to the metal ion or the dithiolene ligands [11].

**Figure 3.**
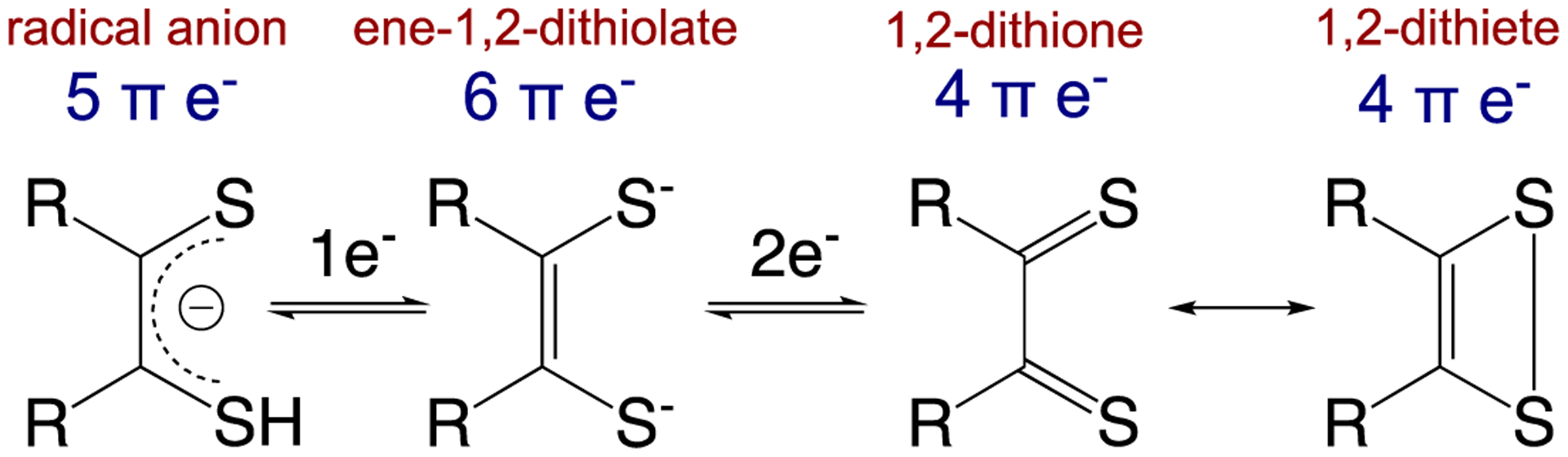
Dithiolene redox states and resonance structures for the oxidized dithione/dithiete forms. (Adapted with permission from *Inorganic Chemistry*, 2016, 55, 785–793. Copyright (2016) American Chemical Society).

Importantly, these non-innocent dithiolene ligands can modulate the nature of the covalent bonding with transition metal ions via the various redox states accessible to the dithiolene (Figure 3) [13]. The ene-1,2-dithiolate is the reduced form of the ligand and possesses six π-electrons. This ligand form is both a σ-donor and π-donor that usually forms strong covalent bonds with an oxidized transition metal ion, as is observed in the active sites of most pyranopterin Mo and W enzymes (e.g., Mo(V)/Mo(VI)-dithiolene bonds). The radical anion form with five π-electrons is usually found in molecules chelated by multiple dithiolene ligands, where extended delocalization of the π-electrons and mixed-valency assists in the stabilization of the metal-ligand bonds. The fully oxidized 1,2-dithione form of the ligand possesses only four π-electrons and can be described by two resonance structures (e.g., the 1,2-dithione and 1,2-dithiete). The low-lying empty π* orbitals of the S=C bonds in the dithione can accept π-electron density from electron-rich low-valent transition metals [16,17], thereby stabilizing such compounds. However, dithione-containing low-valent metal complexes are encountered much less frequently than high-valent transition metal ions coordinated by reduced forms of dithiolene ligands.

In 1982, Johnson and Rajagopalan proposed that Moco consisted of the Mo ion coordinated by the dithiolene fragment of the PDT (Figure 1), from the results of an elegant series of degradative, analytical and spectroscopic studies of sulfite oxidase [26]. This proposed structure was subsequently confirmed by X-ray crystallography [27,28], and numerous examples are now known [29]. Molybdenum and tungsten enzymes are the only known examples of dithiolene coordination in biology, and given the “non-innocent” behavior of dithiolene ligands in simple metal compounds, one may ask what role does dithiolene coordination play in molybdenum enzymes? Through a series of examples involving small molecules and enzymes, we will address this important question and how it relates to control of metal-ligand covalency, reduction potentials, and reactivity in pyranopterin Mo and W enzymes.

## 2. Mo-Dithiolene Bonding

### 2.1. Early Descriptions of Mo-Dithiolene Bonding

**Figure 4.**
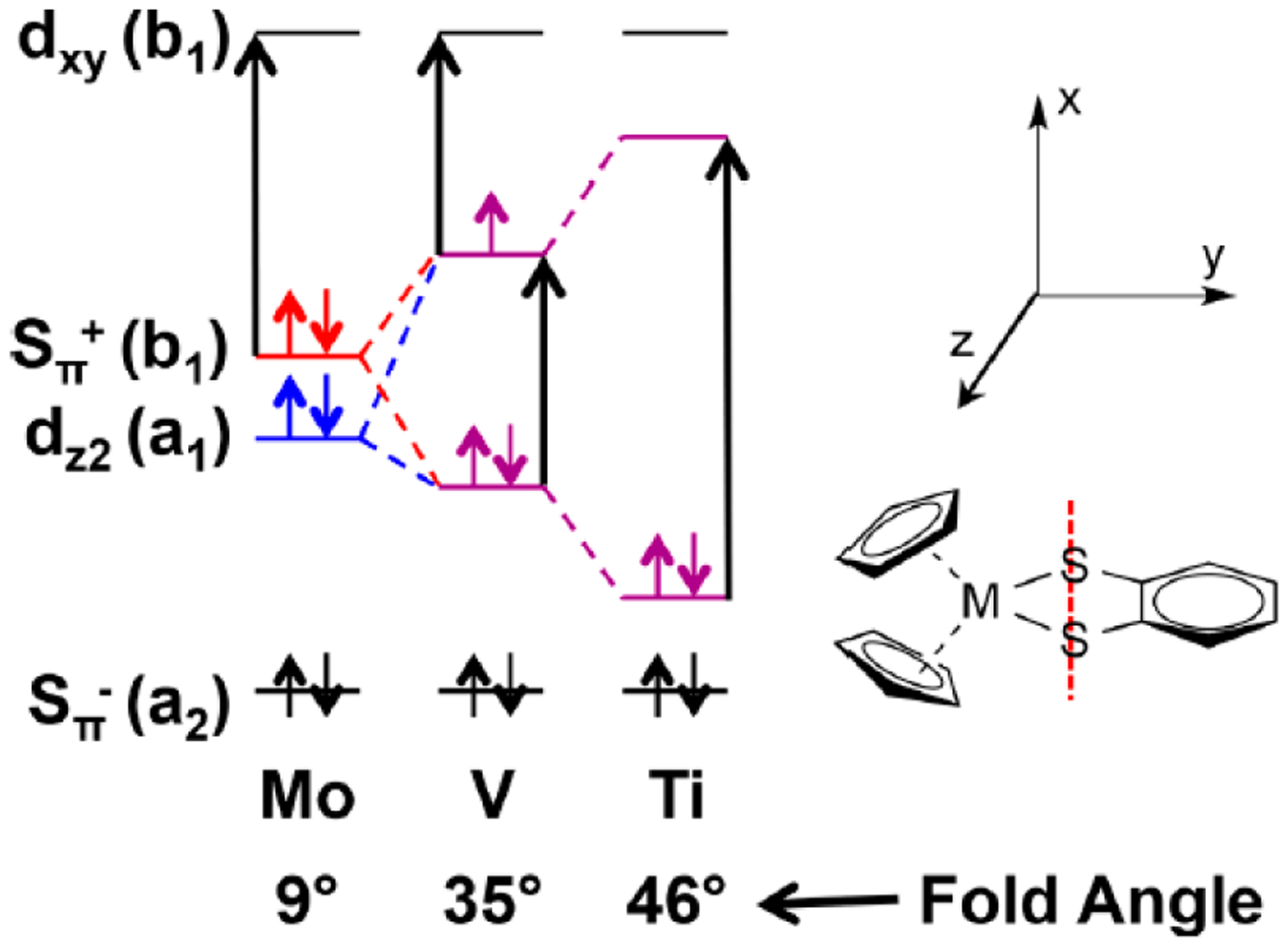
Fold angle distortions as a function of redox orbital electron occupancy in a series of Cp_2_M^IV^(bdt) complexes. (Adapted with permission from *J. Am. Chem. Soc.* 2018, 140, 14777–14788. Copyright (2018) American Chemical Society).

**Figure 5.**
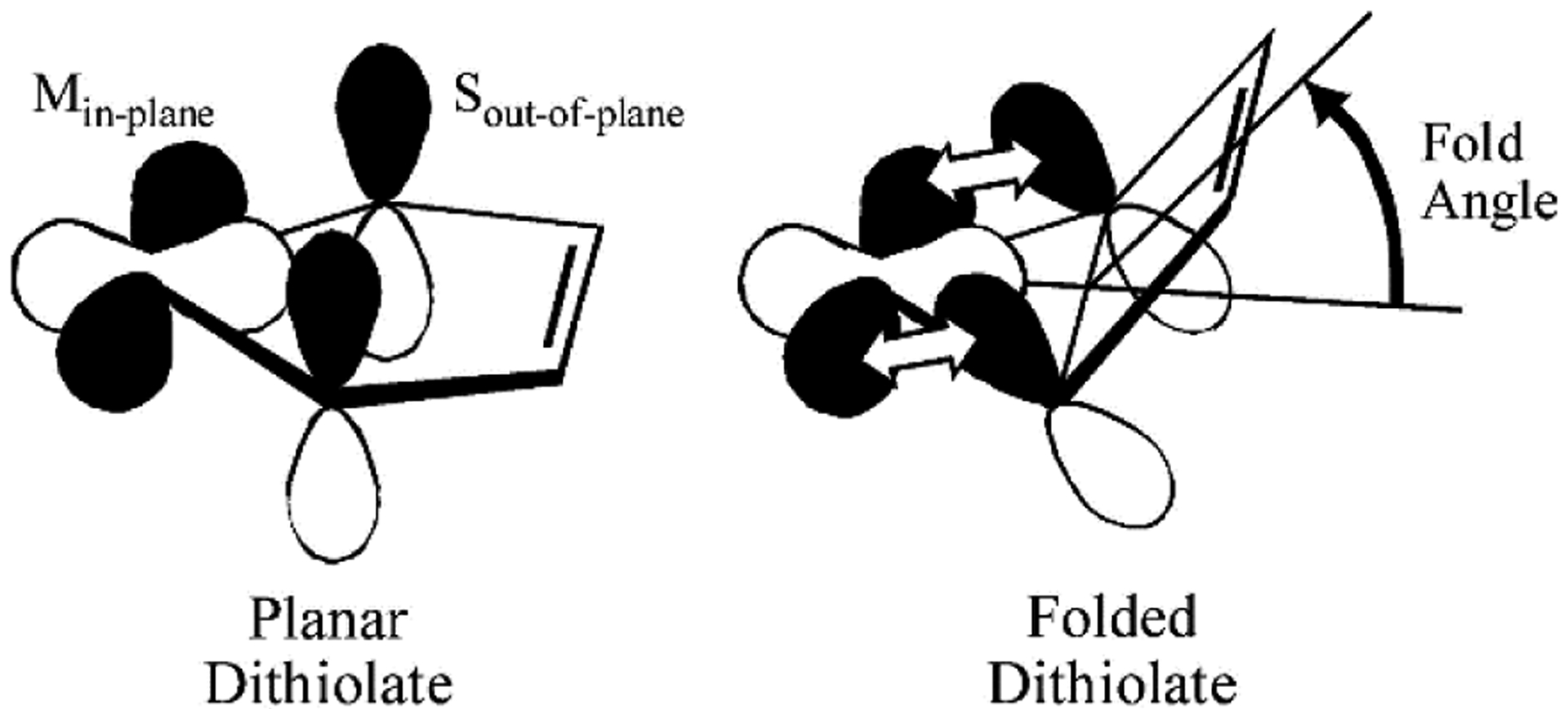
Pictorial description of how the ligand fold angle modulates the degree of mixing between the dithiolene out-of-plane S orbitals (S_π_^+^) and the in-plane Mo(xy) redox orbital. The chelate ring fold is along the dithiolene S–S vector. (Adapted with permission from *Proc. Natl. Acad. Sci. USA.* 2003, 100, 3719–3724. Copyright (2003) National Academy of Sciences.

Some insight into the role of dithiolene coordination in enzymes is provided by the organometallic compounds of the general formula Cp_2_M(bdt), where Cp is C_5_H_5_^−^, and M is either Mo, V or Ti. The fold angle of the dithiolene ligand depends on the formal d-electron count of the metal, and this angle ranges from nearly planar (9°) for Mo (d^2^), to 35° for V (d^1^), and 46°for Ti (d^0^) (Figure 4). Lauher and Hoffman [30] related this increase in the fold angle with decreased d-electron count to donation from the filled out-of-plane S_π_^+^ orbital to the in-plane metal d-orbital (Figure 5). For the molybdenum enzymes, these model compound results imply that the Mo-dithiolene fold angle in Moco could be related to the formal oxidation state of the Mo atom, with Mo(VI) (d^0^) sites possessing a relatively large fold angle and Mo(V) (d^1^) and Mo(IV) (d^0^) sites possessing smaller fold angles. Accurate fold angles are difficult to determine for large protein molecules, but values ranging from 6–33° have been calculated for various molybdenum enzymes [31]. The binding of substrate or inhibitors, and/or dynamic conformational changes in the protein, are expected to modulate the active site chelate fold angle and thereby affect enzyme reactivity [4,32].

### 2.2. Spectroscopic Investigations of Mo-Dithiolene Bonding

#### 2.2.1. Electron Paramagnetic Resonance (EPR) Spectroscopy

**Figure 6.**
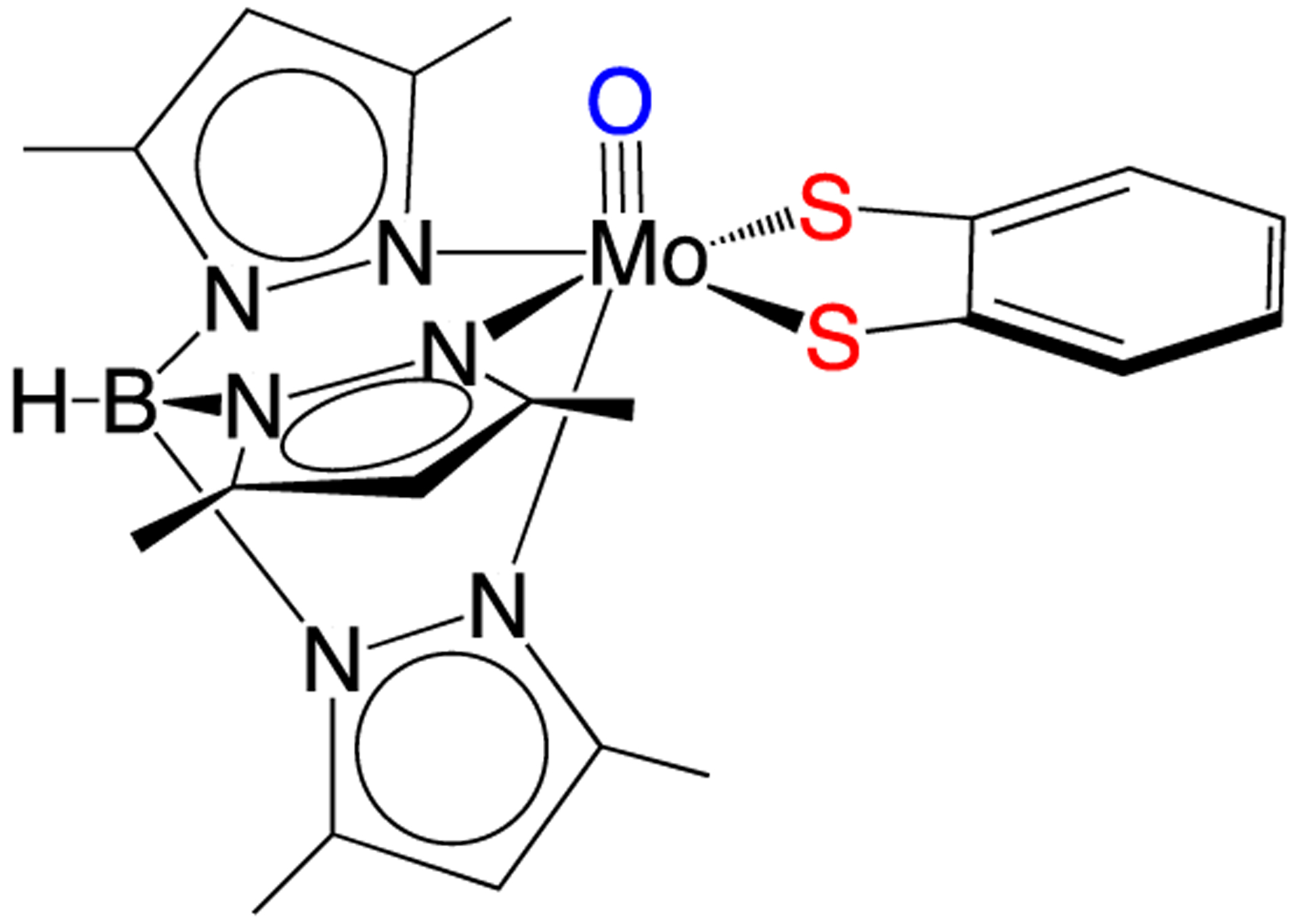
The Tp*Mo^V^O(bdt) model. Note that the apical oxo ligand can be changed to a terminal sulfido or nitrosyl to probe the electronic structure of the Mo-dithiolene unit. The bdt ligand can also be conveniently interchanged with a large variety of other dithiolenes.

An important spectroscopic signature of molybdenum enzymes, such as xanthine oxidase and sulfite oxidase, is a unique Mo(V) electron paramagnetic resonance (EPR) spectrum. The EPR spectra of the enzymes display a relatively large average g-value (g_ave_ = 1.97) and relatively small ^95,97^Mo hyperfine interactions (*hfi*) compared to the EPR spin-Hamiltonian parameters from typical inorganic Mo(V) complexes that possess hard N, O, and Cl donor ligands. The unique EPR parameters for molybdenum enzymes have been ascribed to covalent delocalization of electron density between the Mo(V) center and the sulfur atoms of the coordinated pyranopterin dithiolene unit [33]. The oxo-Mo(V) model compound Tp*MoO(bdt) (Figure 6, where Tp* is hydrotris-(3,5-dimethyl-1-pyrazolyl)borate and bdt is 1,2-benzenedithiolate)) displays Mo(V) EPR spin-Hamiltonian parameters that are very similar to those observed in the enzymes. This supports the proposal of dithiolene coordination in Mo enzymes [34], which has been confirmed by X-ray crystal structures [2]. Recent multidimensional variable frequency pulsed EPR studies of sulfite oxidase, where the sulfur atoms of Moco have been isotopically labeled with ^33^S (I = 3/2), have provided *direct* experimental evidence for delocalization of Mo(V) spin density onto the S atoms of the dithiolene fragment of Moco [35,36]. Density functional theory (DFT) computations show spin polarization effects and strong covalent intermixing between the in-plane metal d_xy_ orbital and out-of-plane p_z_ orbitals of the PDT dithiolene S atoms, which provide a mechanism for the observation of a significant ^33^S hyperfine interaction [12,36].

#### 2.2.2. Electronic Absorption and Resonance Raman Spectroscopies

**Figure 7.**
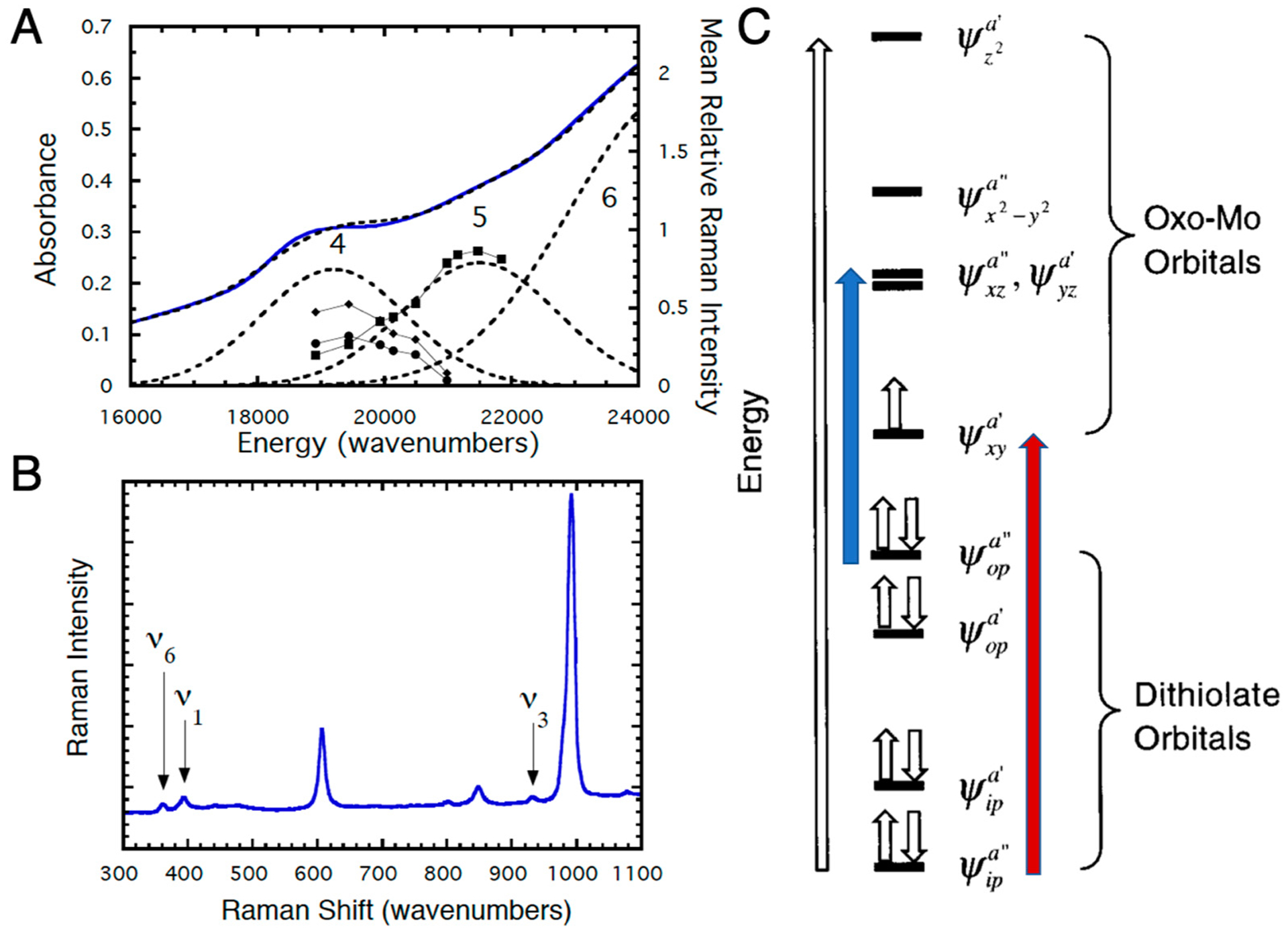
(**A**) Solid state resonance Raman profiles and 5K mull electronic absorption spectrum for Tp*Mo^V^O(bdt). (**B**) Resonance Raman spectrum for Tp*Mo^V^O(bdt) (293K) using 514.5 nm excitation (75 mW). (**C**) General molecular orbital diagram for Tp*Mo^V^O(dithiolene) complexes. The z-axis is oriented along the Mo≡O bond and the energies of the molecular orbitals are not drawn to scale. Transitions are described in the text. (Adapted with permission from *Inorganic Chemistry*, 1999, 38, 1401. Copyright (1999) American Chemical Society).

Experimental investigation of the electronic structures of the Mo centers of enzymes is difficult because of the intense absorptions from other chromophores (e.g., the *b*-type heme in sulfite oxidase and iron sulfur centers and FAD in xanthine oxidase) [37–41]. However, the effects of dithiolene coordination on electronic structure have been investigated for model oxo-Mo(V) compounds (Figure 6) by electronic absorption, XAS, magnetic circular dichroism (MCD), and resonance Raman (rR) spectroscopies [12,14–17,32,33,42–51]. For Tp*MoO(bdt), the electronic absorptions at 19,400 cm^−1^ (Band 4) and 22,100 cm^−1^ (Band 5) are assigned to S → Mo charge transfer bands (Figure 7A) [12]. These assignments have been confirmed by rR spectroscopy (Figure 7A,B), which shows three resonantly enhanced vibrations at 362.0, 393.0, and 931.0 cm^−1^. The lower frequency vibrations (ν_1_ and ν_6_) can be assigned to symmetric S-Mo-S stretching and bending vibrations, and the 931.0 cm^−1^ frequency (ν_3_) is primarily the Mo≡O stretch. Figure 7C shows a molecular orbital diagram that is consistent with the spectroscopic data of Figure 7A,B. Band 5 of Figure 7A is assigned as *ψ*_op_^a″^ → *ψ*_xz_^a″^, *ψ*_yz_^a′^ (blue arrow, Figure 7C), a transition which formally results in the promotion of an electron from an out-of-plane dithiolene molecular orbital to the nearly degenerate Mo d_xz_,_yz_-based orbitals, which are strongly antibonding with respect to the apical Mo≡O bond. This band assignment is supported by the rR enhancement of ν_3_ (squares) with excitation into Band 5 (Figure 7A,B). The preferential enhancement of vibrations ν_1_ (diamonds) and ν_6_ (circles) upon excitation at 514.5 nm (Figure 7A,B) supports assignment of Band 4 as the electronic transition *ψ*_ip_^a″^ → *ψ*_xy_^a′^ (red arrow, Figure 7C), which promotes an electron from the antisymmetric in-plane dithiolene orbital (*ψ*_ip_^a″^) to the half-filled in-plane Mo d_xy_ (*ψ*_xy_^a′^) orbital. The intensity of this electronic transition illustrates the covalency of in-plane metal-dithiolene bonding and suggests that such a pseudo-σ-mediated process could play a role in one-electron transfer steps of enzyme catalysis.

## 3. Synergistic Interactions between the Dithiolene and Pterin Components of the PDT

**Figure 8.**
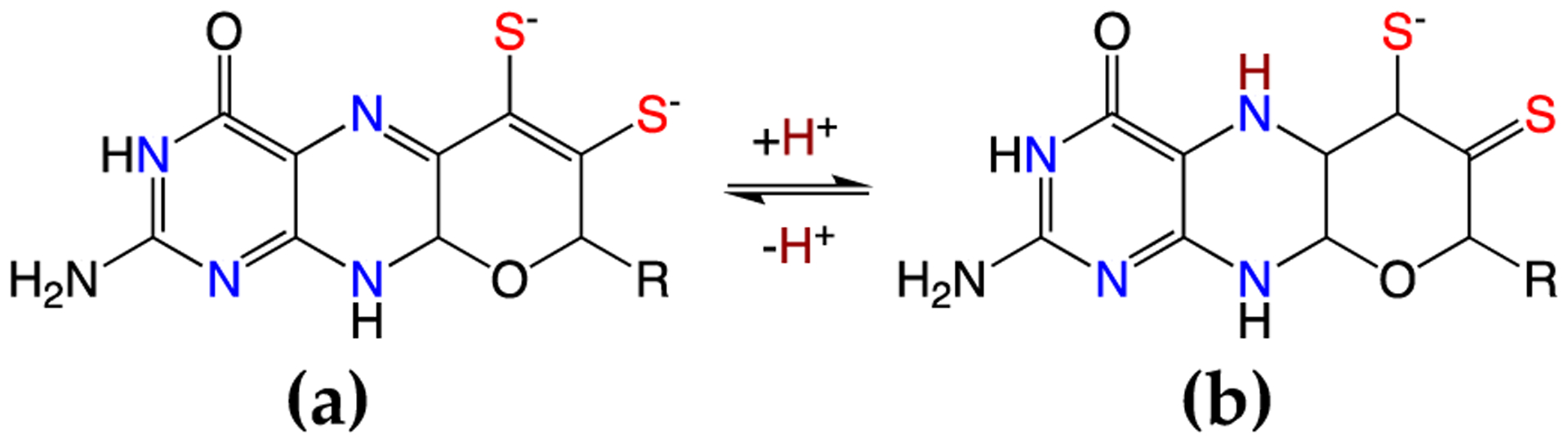
Oxidized PDT ligands: dihydropyranopterin (**a**) and protonated dihydropyranopterin (**b**) yielding the thiol/thione.

Electronic coupling between the dithiolene and the pterin components of the PDT is most prevalent in the dihydropyranopterin form of the PDT [4,15,20,29,52]. This coupling is dramatically reduced in a tetrahydropyranopterin due to the loss of extended π-conjugation in these systems. Two-electron oxidation of the tetrahydro pyranopterin component of the PDT can result in an unusual asymmetric dithiolene known as the “thiol-thione” form that leads to bond and electronic asymmetry in the metal-dithiolene core [4,15,52]. As depicted in Figure 8, the two-electron oxidized 10,10a-dihydropyranopterin can undergo an induced internal redox reaction upon protonation at the N-5 position that involves a subsequent charge transfer between the dithiolene chelate and the piperazine ring of the pterin. This protonation results in a dominant monoanionic thiol-thione chelate form of the ligand when bound to Mo or W. This thiol-thione character can also occur in the absence of protonation by the concept of resonance, which may also be described as configurational mixing between the thiol-thione and dithiol states. This type of thiol-thione chelate has been observed and studied in a small molecule Mo(IV) systems [4,15,20,52]. In these systems, excited state thiol-thione character was shown to be admixed into the ground state configuration using a variety of spectroscopic and computational probes of the electronic structure. The analysis of the data indicates that a two-electron oxidized pterin is inherently electron withdrawing, allowing for a low-lying dithiolene → pterin intraligand charge transfer (ILCT) state to mix with the ground state to provide a variable degree of thiol-thione character in the electronic ground state.

Definitive spectroscopic signatures are associated with the presence of a dihydropterin form of the PDT ligand. It is observed that the dithiolene → pterin intraligand charge transfer (ILCT) band is intense (E = 20,000–27,500 cm^−1^; ε ~ 10,000–16,000 M^−1^ cm^−1^) [52], and there is considerable resonance enhancement of numerous Raman vibrations that can be assigned as originating from pterin and dithiolene C = C and C = N vibrations. Key resonance enhanced vibrational modes that can be used to characterize the presence of dihydropterin thiol-thione character in the enzymes include the 1508 cm^−1^ and 1549 cm^−1^ pyranopterin–dithiolene stretching frequencies that were observed in this Mo(IV) cyclized pyranopterin dithiolene model compound. This oxidized pyran ring closed form of the PDT has yet to be definitively observed in any pyranopterin Mo enzyme, but its presence would have profound implications on the electronic structure of the Mo site. Namely, the change in ligand charge from −2 to −1 leads to an asymmetric reduction in the charge donated by the monoanionic ligand compared to the dianionic dithiolene. Charge effects on oxygen atom transfer catalysis have recently been explored in model compounds showing dramatic rate enhancements in the oxidative half reaction that leads to substrate reduction [53]. This reactivity correlates with a large shift in the Mo(VI/V) reduction potential between cationic [Tpm*MoO_2_Cl]^+^ (−660 mV vs. Fc^+^/Fc) and charge neutral Tp*MoO_2_Cl (−1010 mV vs. Fc^+^/Fc) [53]. The same effect on redox potential and reactivity would be expected in enzymes that could adopt an oxidized PDT with a thiol-thione configuration. The presence of a thiol-thione form of the PDT in an enzyme would also have a considerable impact on the active site electronic structure, and enable the pyranopterin to play a more significant role in catalysis by fine-tuning the Mo redox potential and providing a π-pathway for electron transfer regeneration of the active site [52]. Additionally, the asymmetry in the dithiolene (thiol/thione) charge donation would be expected to result in a significant *trans* effect or *trans* influence on oxo or sulfido ligands that are coordinated to the Mo or W ion and oriented *trans* to the thione sulfur.

## 4. The Electronic Buffer Effect and Fold Angle Distortions

### 4.1. Photoelectron Spectroscopy (PES) Studies

A common structural feature of the large group of pyranopterin Mo enzymes that catalyze a wide range of oxidation/reduction reactions in carbon, sulfur, and nitrogen metabolism is coordination by the sulfur atoms of one (or two) unique dithiolene groups derived from the side chain of a novel substituted pterin (PDT, Figure 1). Given the electronic lability of the dithiolene, a possible role of dithiolene coordination in molybdoenzymes is to buffer the influence of other ligands and changes in the formal oxidation state of the metal. Gas-phase photoelectron spectroscopy (PES) is a powerful tool for probing metal-ligand covalency in isolated molecules. Gas-phase ultraviolet PES of the molybdenum model complexes with the general formula Tp*MoE(tdt) (Figure 6, where E = O, S, or NO, and tdt = 3,4-toluenedithiolate), exhibit nearly identical first ionization energies (6.88–6.95 eV) even though there is a dramatic difference in the electronic structure properties of the axial ligand. Collectively, these results have provided direct experimental evidence for the “electronic buffer” effect of dithiolene ligands [54].

Additional evidence for the electronic buffer effect of dithiolene ligands has been provided by gas-phase core and valence electron ionization energy measurements of the series of molecules Cp_2_M(bdt) (Figure 4, Cp = η^5^-cyclopentadienyl, M = Ti, V, Mo, and bdt = benzene-1,2-dithiolate). Comparison of the gas-phase core and valence ionization energy shifts provides a unique quantitative energy measure of valence orbital overlap interactions between the metal and the sulfur orbitals that is separated from the effects of charge redistribution. The results explain the large amount of sulfur character in the redox-active orbitals and the electronic buffering of oxidation state changes in metal-dithiolene systems. The experimentally determined orbital interaction energies also reveal a previously unidentified overlap interaction of the predominantly sulfur HOMO of the bdt ligand with the filled π orbitals of the Cp ligands, suggesting that direct dithiolene interactions with other ligands bound to the metal could be significant for other metallodithiolene systems in chemistry and biology [55].

### 4.2. A Large Fold Angle Distortion in a Mo(IV)-Dithione Complex

**Figure 9.**
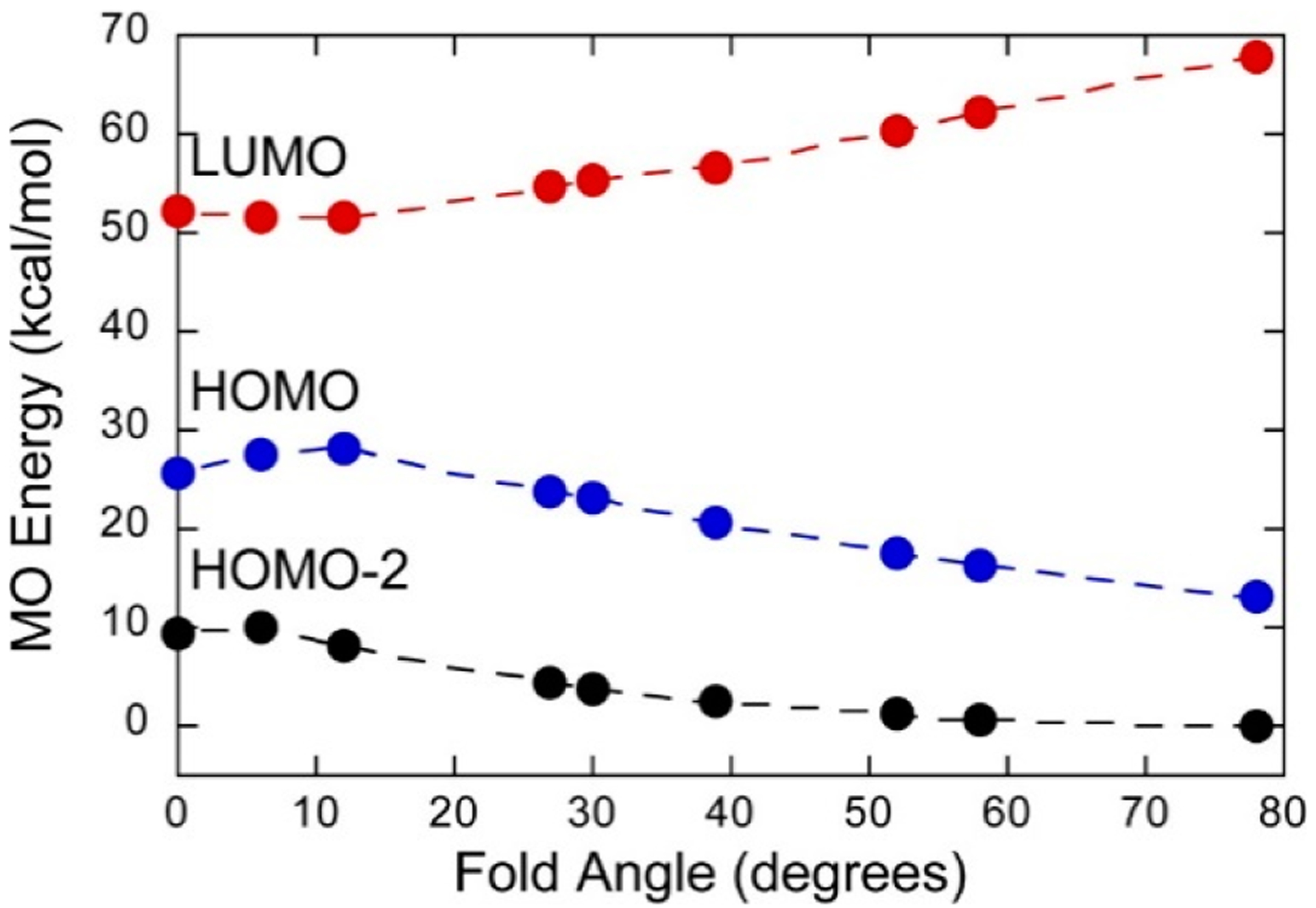
Frontier orbital energies as a function of fold angle in Mo^IV^O(SPh)_2_(^i^Pr_2_Dt^0^), which possesses a dithione π-acceptor ligand. (Adapted with permission from *Inorganic Chemistry*, 2016, 55, 785–793. Copyright (2016) American Chemical Society).

**Figure 10.**
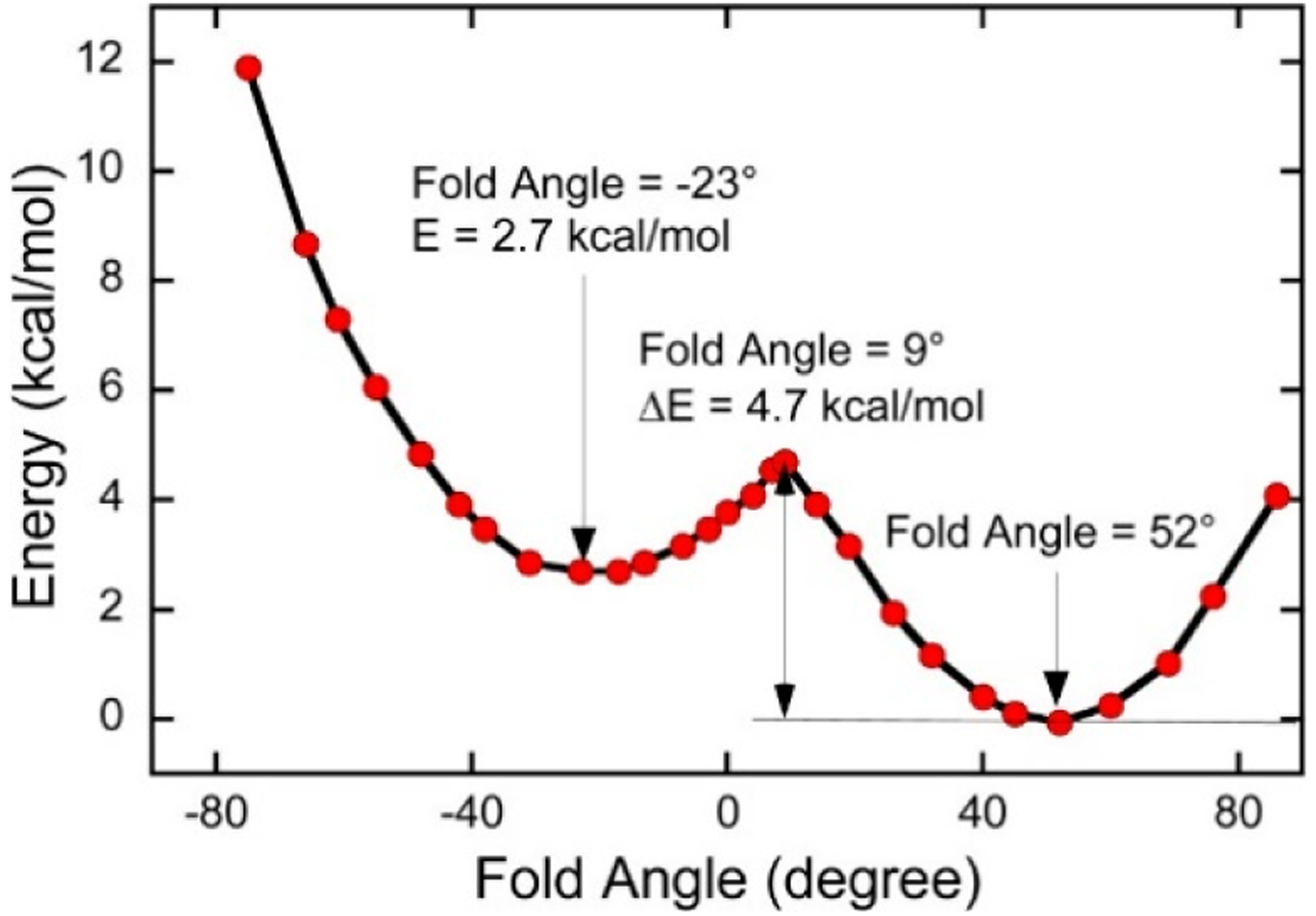
A double well in the ground state potential energy surface of Mo^IV^O(SPh)_2_(^i^Pr_2_Dt^0^) as a function of the ligand fold angle. (Adapted with permission from *Inorganic Chemistry*, 2016, 55, 785–793. Copyright (2016) American Chemical Society).

Mo(IV)-dithione complexes are much rarer than Mo(V)/Mo(VI)-dithiolene complexes. Recently, a detailed spectroscopic and computational study was performed on a novel Mo(IV)-dithione complex, MoO(SPh)_2_(^i^Pr_2_Dt^0^) (where ^i^Pr_2_Dt^0^ = *N*,*N*′-isopropylpiperazine-2,3-dithione) [17]. The structure of this unusual molecule was determined by x-ray crystallography and displays a remarkably large dithiolene fold angle (η = 70°). This large fold angle was compared to that observed in more than 75 other metallodithiolene complexes found in the Cambridge crystallographic database, where fold angles were found to range from 0.3° to 37.3° with an average value for η of 12.5° [17]. The large fold angle distortion in the metallodithiolene ring of MoO(SPh)_2_(^i^Pr_2_Dt^0^) is reflected in its unusual electron absorption spectrum. The combination of an electron rich Mo(IV) center and electron donating thiolate (SPh) ligands results in the presence of low-energy Mo(IV) d(x^2^-y^2^) → dithione MLCT and thiolate → dithione LL’CT transitions as a result of the strong π-acceptor character of the dithione ligand. These spectral assignments are supported by resonance Raman profiles constructed for the 378 cm^−1^ S-Mo-S symmetric stretch and the 945 cm^−1^ Mo≡O stretch in addition to the results of TDDFT computations. The donor-acceptor nature of the complex was revealed in a molecular orbital fragments analysis using a donor fragment, [(PhS)_2_Mo(IV)]^2+^ (F1) and an acceptor fragment, [^i^Pr_2_Dt^0^] (F2). The analysis showed that 21% of the F1 HOMO was mixed into the F2 fragment LUMO at a 70° fold angle. In contrast, only 5% of the F1 HOMO was mixed into F2 fragment LUMO in a planer configuration (η = 0°), correlating the effective π-acceptor ability of the dithione with the ligand fold angle. The effects of this HOMO-LUMO mixing also affects the HOMO-LUMO gap, with the HOMO-LUMO gap increasing at larger fold angles (Figure 9). The increased covalency that results from the fold angle distortion represents an example of a strong pseudo-Jahn-Teller effect, vide infra, involving vibronic coupling between the ground state and a low-energy excited state in the non-distorted (η = 0°) geometry of this molecule. A scan of the potential energy surface as a function of this fold angle distortion coordinate results in an asymmetric double well potential (Figure 10), with the global minimum representing a ground state geometry with the dithione ligand fold distorted toward the apical oxo ligand. Thus, an oxidized dithione form of the PDT present in an enzyme active site would be expected to possess a very large ligand fold angle, unless the polypeptide enforces a more planer fold angle geometry.

### 4.3. Low-Frequency Pyranopterin Dithiolene Vibrational Modes in Xanthine Oxidase/Dehydrogenase

Low-frequency dithiolene distortions that are coupled to large electron density changes at the Mo ion represent an example of the electronic buffer effect [54], and have been probed in bovine xanthine oxidase (XO) and *R. capsulatus* xanthine dehydrogenase (XDH) using resonance Raman spectroscopy [40]. Computations have shown that exciting into a low-energy Mo(IV) → product metal-to-ligand charge transfer (MLCT) band results in a large degree of change transfer from the Mo(IV) HOMO to the product LUMO, resulting in an excited state with significant Mo(V) hole character (e.g., Mo(IV)–P^0^ → Mo(V)–P·). Thus, the optical charge transfer process mimics the instantaneous one-electron oxidation of the Mo ion, which is encountered in the electron transfer reactions of the enzymes.

**Figure 11.**
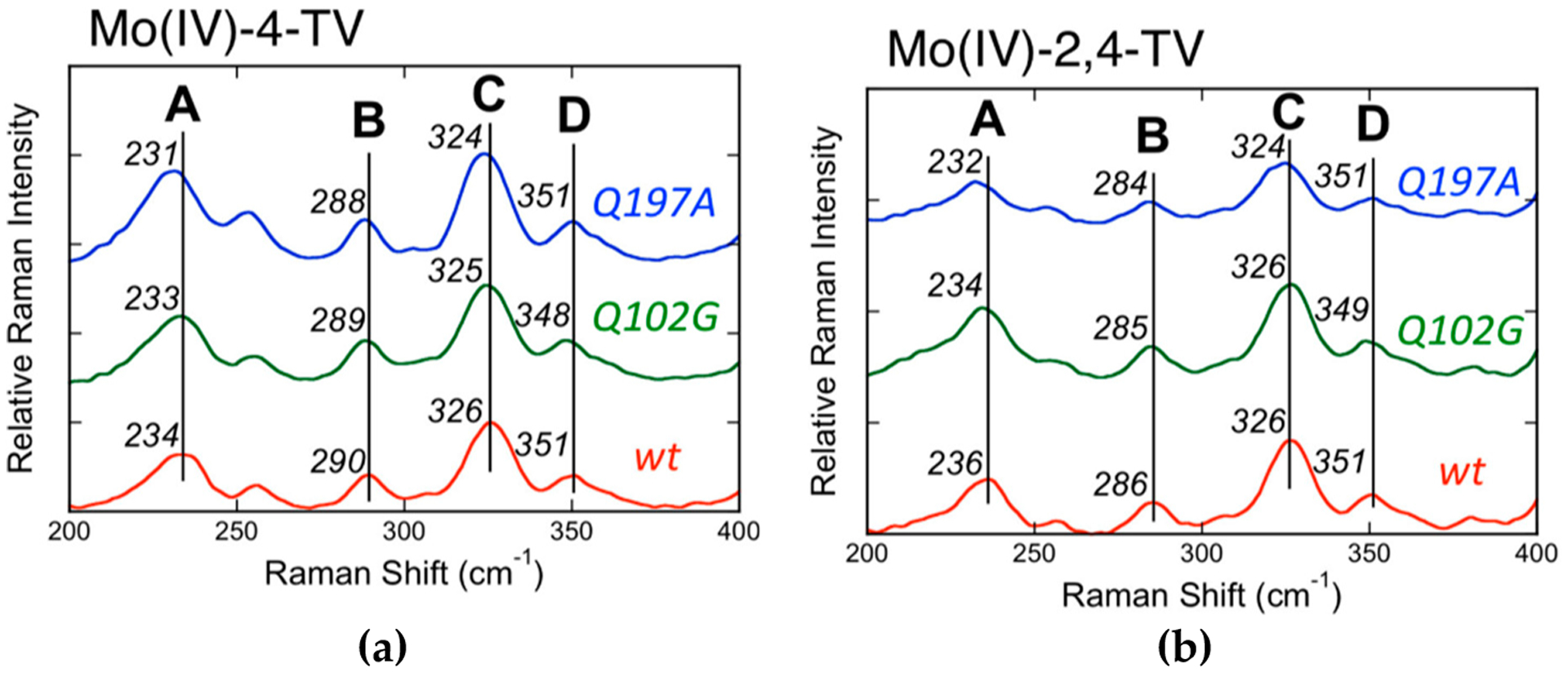
Low-frequency rR spectra for wt, Q102G, and Q197A XDH, Mo^IV^–4-TV (**a**) and Mo^IV^–2,4-TV (**b**). Raman spectra were collected on resonance with the Mo(IV) → P MLCT band using 780 nm laser excitation (Adapted with permission from *Inorganic Chemistry*, 2017, 56, 6830–6837. Copyright (2017) American Chemical Society).

The Mo(IV) → 2,4-TV and Mo(IV) → 4-TV (2,4-TV = 2,4-thioviolapterin; 4-TV = 4-thioviolapterin) MLCT bands are red-shifted relative to the Mo(IV) → violapterin MLCT band [39,40,56–59]. The red-shift of the Mo^IV^–2,4-TV and Mo^IV^–4-TV MLCT bands eliminates spectral overlap with the absorption envelope of the 2Fe–2S spinach ferredoxin clusters and FAD. The elimination of the FAD fluorescence background and spurious signals deriving from 2Fe–2S vibrations contributing to the Raman spectrum allow for the acquisition of very high-quality resonance Raman data. Multiple low-frequency (200–400 cm^−1^) Raman vibrations are observed to be enhanced when using laser excitation on resonance with the Mo(IV) → product MLCT band [40], and these have been assigned as a vibrational mode involving dithiolene folding, Mo≡O rocking, and pyranopterin motions (Band A: Mo^IV^–4-TV = 234 cm^−1^; Mo^IV^–2,4-TV = 236 cm^−1^), a ring distortion vibration that possesses both Mo-SH and pyranopterin motions (Band B: Mo^IV^–4-TV = 290 cm^−1^; Mo^IV^–2,4-TV = 286 cm^−1^), the symmetric S-Mo-S dithiolene core stretching vibration (Band C: Mo^IV^–4-TV = 326 cm^−1^; Mo^IV^–2,4-TV = 326 cm^−1^), and the corresponding asymmetric S-Mo-S dithiolene stretch (Band D: Mo^IV^–4-TV = 351 cm^−1^; Mo^IV^–2,4-TV = 351 cm^−1^) (Figure 11). Thus, the instantaneous generation of a hole on the Mo center (Mo(IV)–P^0^ → Mo(V)–P·) by photoexcitation is felt by the dithiolene chelate and extends all the way to the amino terminus of the PDT. The most resonantly enhanced mode in this spectral region is Band C, the symmetric S-Mo-S dithiolene core stretching, and the frequency of this mode and Band D are similar to those observed in Tp*MoO(bdt) [12,32], which were assigned as the chelate ring symmetric S-Mo-S stretching and bending vibrations, respectively. Band A is significant, since it possesses dithiolene ring folding character indicating that electron density changes at Mo are buffered by a distortion along this low-frequency coordinate, as has been observed in the various model systems described in this review. These observations strongly support an electron transfer role for the PDT in catalysis, with the dithiolene contributing to the Mo-S covalency necessary for increasing the electronic coupling matrix element for electron transfer (*H*_*DA*_) and to affect the Mo reduction potential via the covalency in the Mo–S_dithiolene_ bonds.

## 5. Vibrational Control of Covalency

A combination of MCD, electronic absorption, electron paramagnetic resonance, resonance Raman, and photoelectron spectroscopies has been used in conjunction with theory to reveal vibrational control of metal-ligand covalency in a series of Cp_2_M(bdt) complexes (M = Ti, V, Mo; Cp = η^5^-C_5_H_5_) [60] (Figure 4). The work is important because it has allowed for a detailed understanding of how redox orbital electron occupancy (Ti(IV) = d^0^, V(IV) = d^1^, Mo(IV) = d^2^,) affects the nature of the M-dithiolene bonding scheme at parity of the ligand set and at parity of charge. In this series of complexes, large changes in the metallodithiolene fold angle and electronic structure are observed as electrons are successively removed from the redox orbital (Figure 4). These electron occupancy effects on the fold angle distortion are now understood in terms of the pseudo-Jahn-Teller effect (PJT). PJT-derived molecular distortions originate from the mixing of the electronic ground state (Ψ_0_) with specific excited states (Ψ_i_) [61,62]. The ground state-excited state energy gap (2Δ), the matrix elements (*F*_0i_) of the vibronic contribution to the force constant (*F*), and the primary non-vibronic force constant (*K*_0_) all govern the degree of the ligand fold distortion according to:

(1) $$F_{0i}=\langle \psi_{0}\left|\frac{\partial H}{\partial Q}\right|\psi_{i}\rangle$$

(2) $$F^{2}>\Delta \cdot K_{0}$$

**Figure 12.**
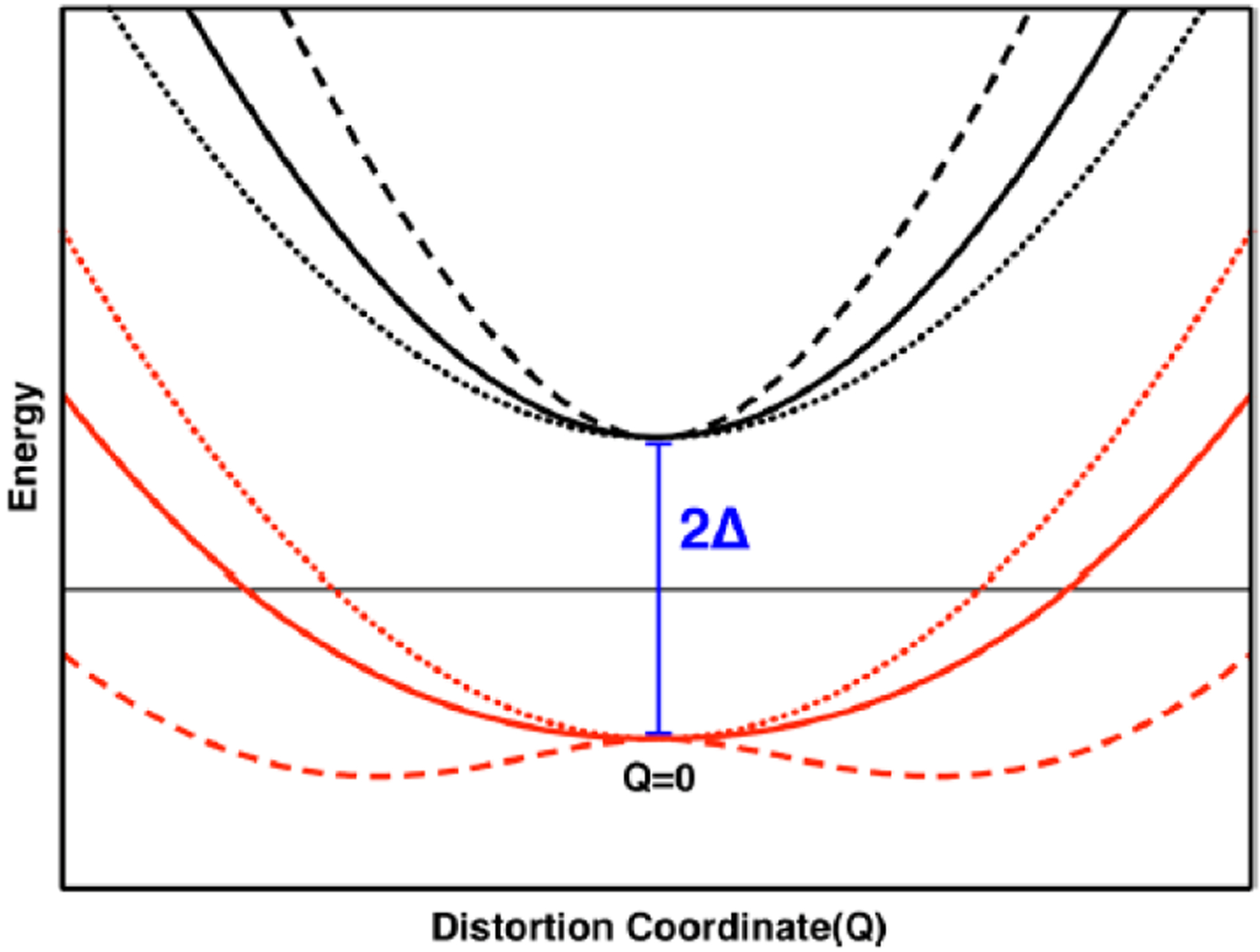
The excited state (black) and ground state (red) potential energy surfaces associated with varying values of F^2^ (Dotted: F^2^ = 0, solid: F^2^ = Δ·K0, dashed: F^2^ = 2Δ·K0) When the condition F^2^ > Δ·K0 is met one observes that the single-well ground state potential energy surface distorts into a double-well potential. The F^2^ > Δ·K0 criteria describe a strong PJT effect. (Adapted with permission from *J. Am. Chem. Soc.* 2018, 140, 14777–14788. Copyright (2018) American Chemical Society).

At the critical threshold defined by Equation (2), the metallodithiolene centers of Cp_2_M(bdt) can distort along the dithiolene fold angle coordinate to yield a double well potential energy surface (Figure 12), and the magnitude of the PJT distortion is maximized by a large *F*, a small Δ, and a small *K*_0_. Thus, the PJT distortion in these Cp_2_M(bdt) complexes effectively couples soft fold angle bending modes in the M-dithiolene chelate ring to the inherent electronic structure of the system via the d-electron count. Importantly, the mixing of low-energy charge transfer states into the ground state by the PJT effect controls the covalency of the M-S bonds.

One of the unique aspects of Mo-S and W-S bonding is the small energy gap between filled dithiolene-based orbitals and the lowest energy metal-based orbital, which naturally leads to low-energy charge transfer states that can mix with the ground state. Mode softening along the dithiolene fold coordinate is important in pyranopterin Mo and W enzymes since this leads to a potential energy surface where a large range of dithiolene fold angles may be sampled without paying a prohibitive energy penalty. This effect is maximized when *F*^2^ ≅ Δ·*K*_0_. Thus, a low-energy pathway is operative that can minimize energetically unfavorable reorganizational energy contributions along the reaction coordinate, which accompany redox changes at the metal ion. As mentioned previously, these fold angle distortions have been shown to be kinematically coupled to low frequency pyranopterin modes in XO and contribute to low-energy barriers for electron transfer regeneration of the active site. However, in the enzymes there may be either a competing or additive relationship between active site distortions that are driven via the d-electron count of the metal ion and distortions that are imposed by the protein. Vibronic coupling effects that derive from different occupancy numbers for the redox-active orbital will function to modulate the enzyme reduction potential in the oxidative and reductive half reactions of pyranopterin Mo and W enzymes, and this occurs by modulating the degree of metal-ligand covalency via low-frequency distortions at the active site.

## 6. Conclusions

This review focuses on the electronic structures, molecular structures, and spectroscopic properties of well-characterized metallodithiolene compounds in order to provide deep insight into the role(s) of metal-dithiolene bonding in pyranopterin dithiolene containing enzymes (Figure 1). The discovery, in the early 1960s, that transition metal dithiolene compounds undergo a series of one-electron oxidation-reduction reactions (Figure 2), provided the first evidence for the “non-innocence” of dithiolene ligands and the highly covalent nature of metal-dithiolene bonding. Additional links between metal-dithiolene covalency and electronic and molecular structure were posited from theoretical studies of bent metallocene-dithiolene compounds (Figure 4) by Lauher and Hoffman in 1976 [30], who related metal-dithiolene chelate ring “folding” with the metal ion d-electron configuration. Investigations of Mo dithiolene compounds by electronic absorption, resonance Raman, and EPR spectroscopies showed that S → Mo charge transfer bands dominate the visible spectrum and that there is substantial delocalization of spin density onto the S atoms of the dithiolene. Recent comprehensive studies of bent metallocene-dithiolene compounds have shown that low-energy ligand fold distortions arise from a pseudo-Jahn-Teller (PJT) effect, which involves vibronic coupling of the electronic ground state with electronic excited states to control metal-ligand covalency [60] (Section 5). This vibronic coupling process may play critical roles in the catalytic cycles of pyranopterin Mo and W enzymes by dynamic and/or static modulation of redox potentials and providing a superexchange pathway for electron transfer through the PDT framework. However, greater understanding of how geometric and electronic structure control reactivity, and define function in Mo and W enzymes, will require linking the concepts that have been developed for metallodithiolenes to the emerging results from studies of well-characterized compounds that mimic the pterin component of PDT (Section 3). Exploring the synergistic interactions between the dithiolene and pterin components of the PDT and the metal ion will be challenging, but such research promises to provide important insights into these critically important enzymes.
